# Artificial Intelligence for Healthcare in Africa

**DOI:** 10.3389/fdgth.2020.00006

**Published:** 2020-07-07

**Authors:** Ayomide Owoyemi, Joshua Owoyemi, Adenekan Osiyemi, Andy Boyd

**Affiliations:** ^1^Department of Biomedical and Health Informatics, University of Illinois at Chicago, Chicago, IL, United States; ^2^ZMP Inc., Tokyo, Japan; ^3^Department of Family Medicine, University College Hospital, Ibadan, Nigeria

**Keywords:** Africa, artificial intelligence, digital health, machine learning, sustainable development goals, data

## Introduction

Digital technology will play a significant role in achieving sustainable human development worldwide. In 2015, United Nations Member States set 17 goals, the Sustainable Development Goals (SDGs), to provide a road map for the achievement of Earth's peace and human prosperity by 2030. SDG 3, as one of the goals which is aimed at ensuring healthy lives and promoting well-being for all at all ages, will greatly benefit from the implementation of digital technology. With over a billion people, Africa can be better positioned to surmount its health challenges—especially regarding maternal and child health and infectious and non-communicable diseases—using digital technology including artificial intelligence (AI).

AI is defined as the automation of activities associated with human thinking such as decision-making, problem-solving, and learning (1).

AI was first used in medicine in the 1970s when medical expert systems—based on Bayesian statistics and decision theory—diagnosed and recommended treatments for glaucoma and infectious disease (2). Progress in Bayesian networks, artificial neural networks, and hybrid intelligent systems in the late 1990s has scaled up bioinformatics research, thereby expanding uptake of medical artificial intelligence (MAI) (3). Global investment in MAI is projected to hit about $6.6 billion by 2021 as it is anticipated that AI implementations in healthcare can help save $150 billion in costs by 2026 (4).

At present, a more meaningful application of MAI occurs in developed nations compared with what is obtained in Africa. The United Nations in two different forums has signaled a need to change this narrative by bringing stakeholders together to discuss how AI can be used to deliver critical public services and help in the journey toward achieving the SDGs (5). In this paper, we briefly highlight contemporary MAI use in Africa, along with its opportunities, challenges, and likely prospects.

## Contemporary Artificial Intelligence for Healthcare in Africa

Historically, MAI was piloted in Africa in the mid-1980s. MAI deployed in Kenya improved health worker–patient interaction quality with evidence of increased number of symptoms elicited (6). To improve the detection of common and potentially blinding eye disorders, MAI (successfully implemented in the US) was piloted in Egypt some time in 1986 (7). Similarly, in the Gambia, a probabilistic decision-making system assisted rural health workers to identify life-threatening conditions in outpatient clinics. The MAI performed tolerably well in detecting 88% of cases. Computerized Aid To Treat (CATT) was also used in drug prescriptions in South Africa by nurses based on a cost-and-effectiveness algorithm (8, 9).

In recent times, the healthcare application of AI in Africa has only seen a few pilots and test cases. For example, in South Africa, a multinomial logistic classifier-based system is being applied to human resource planning, especially to predict how long health workers might stay in public service (10). A partnership comprising researchers and a social enterprise has been developing an AI planning application for optimizing the scheduling of community health workers (CHWs) in communities in Africa (11).

In Nigeria, Ubenwa is a start-up that is using signal processing and machine learning to improve the diagnosis of birth asphyxia in low-resource settings (12). Bellemo et al. (13) conducted a study in using AI to diagnose diabetic retinopathy in Zambia which showed significant and promising results when compared with human assessments. It showed clinically acceptable performance in detecting referable diabetic retinopathy (13). The Delft Institute's CAD4TB software has been employed in pilot studies examining the use of a computer-aided diagnosis of pulmonary tuberculosis from chest radiographs in Tanzania and Zambia. The results are exciting as the performance of CAD4TB compared well with that of human experts (14, 15).

Pharmaceutical industries have also benefited from the use of AI in Nigeria. A group of five high school girls developed an app based on an MIT open-source software to identify fake drugs in Nigeria. Their stellar idea received recognition in 2018 upon winning a Silicon Valley contest. Earlier in 2019, Adebayo Alonge, a Nigerian trained pharmacist won the grand prize of Hello Tomorrow Global Challenge 2019 for his AI-hyperspectral platform for authenticating drugs (16, 17).

## Discussion

### Issues Around Implementing Artificial Intelligence for Healthcare in Africa

#### Data Availability and Quality

Machine learning and precision medicine applications require a training dataset for which the outcome variable (e.g., onset of disease) is known. An important challenge in applying AI for health and medicine is the lack of large clinical datasets for training AI models. This is especially true for datasets with labels, which require doctors/medical expert annotation and therefore are costly and time-consuming to collect (18). With the low level of digitization and electronic medical record use across Africa, there is a paucity of locally generated useful data that are important for building AI systems (19).

The concept of algorithmic bias—which implies that AI is as good as the data on which it is trained on—is an important factor in the development of AI products and applications. This factor, though global and not just an African problem, has a more pronounced effect when AI applications are introduced to the African setting (20). Because most AI applications are being developed outside Africa, most datasets available are from people who differ from Africans physiologically. While this might not be a big issue as seen in the study done on diabetic retinopathy, it might affect the sensitivity adversely in other cases (13). AI systems could also contain algorithms that stem from the prejudices and specific beliefs of the creators of the AI systems. This introduces the possibility of unintended bias which creates potential discriminations and wrong outcomes if applied to others in low-resource settings without their developmental input and data (21–23).

#### Legal and Policy Issues

Across the world, a lot of countries are just developing a governance policy or legal framework for the implementation of AI in different sectors including healthcare (24, 25). Some countries in Africa do not have a national digital health policy or strategy which will guide the implementation and monitoring of digital health strategies. African stakeholders also believe that low government engagement at the policy level has stymied adoption, and a stronger engagement will encourage early adoption of AI and other digital tools (26).

In Africa and around the world, there are no enacted laws guiding who takes responsibility for adverse outcomes that might result from the usage of AI in healthcare which is highly likely considering how and where AI might be applied in healthcare. The most probable resolutions might involve the application of existing laws, but some areas and scenarios are not anticipated or covered by the existing laws. This will have legal implications for users and patients in these African countries (22).

#### Costs of Artificial Intelligence Application and Adoption

The cost of assembling an AI/ML-based solution in healthcare is difficult to ascertain in developed countries and could range from as low as $6,000 for simple chatbots to as high as millions of dollars (27). Costs associated with the application or adoption of AI are as follows:

1. Data acquisition and preparation costs: This involves data collection proper and cleaning up and annotation of data. The quality of an AI application is mostly dependent on the quality of data on which it is built. Therefore, this stage is usually critical and time-consuming and can also be very costly depending on the granularity of the information required to be extracted from the collected data. COCO, a large-scale object detection, segmentation, and caption dataset, contains more than 200,000 labeled images containing semantic and instance information of the contents of each image (28). This is a popular dataset for tasks involving pattern recognition in images which are also useful in health science. Even though this dataset is only available for free when used for research purposes, a similar scale of the dataset will be needed for applications that aim at addressing problems in healthcare for Africa. According to price listings by companies such as Scale.ai, annotating a single image will cost up to $6.40 for semantic information. Hence, the cost associated with data acquisition is non-trivial (29).
2. Hardware and computing resources: AI is not a new technology. In fact, back-propagation, the algorithm upon which most powerful AI models are based was first introduced in 1975 (30, 31). The most recent breakthroughs in AI were only possible due to the convergence of powerful computing resources and the availability of massive data. That is, due to the introduction of special computer hardware such as dedicated graphics processing units (GPUs), it has become easier to process the high volume of calculations needed to train and run artificial neural networks faster than it has ever been done. Nowadays, the accuracy of a model can be predicted based on the magnitude of the available data and the computing power.
  Unfortunately, this has also contributed to driving up the cost of developing new models and solutions, even creating new industry sectors such as Amazon Web Services and Microsoft Azure Cloud Computing Services that offer computing in the cloud for time-based fees. Depending on the computing resources needed for each project, the average cost could be up to $25 per hour for a single GPU instance. Hence, a project can be expected to incur running costs ranging from $10,000 to $30,000 by the end of the year (32).
3. System maintenance and upgrading: This is the cost involved in ensuring that an AI model or application keeps working as expected when it is deployed. In practice, this is not always the case. The application, usually, will have to be changed in significant ways for it to keep up with the user expectations. The reason for this is largely due to the rate at which the technology itself changes. Since this is still a continuously growing technology, for research and industry, new tools are introduced more often, and old tools soon become outdated and need to be upgraded. According to the research by Sculley et al. (33), it was found that while machine learning offers a path to quickly engineering complex systems, the convenience comes with tremendous downstream costs.

#### Inadequate Infrastructure

Another major factor limiting the speed of development of this technology in Africa is inadequate infrastructure evident by low Internet penetration (39%) and sociocultural factors impairing adoption (34). There is also widespread inaccessibility to electricity across the continent; about half of Africans have no access to electricity, and this has made it difficult to execute and sustain digital approaches in different sectors of the economy including healthcare (35). Adair-Rohani et al. (36) found out that less than 30% of health facilities on the continent had access to reliable electricity.

Digital health infrastructures—on which some of these MAI solutions may be built or integrated—have also been found to be inadequate (37). Time as an economic good is yet to be fully described in Africa's health space as the period spent designing a solution may hinge on whether it was rented, bought, or built. Some solutions are available off the shelf and require less time to deploy into healthcare, but complex health problems need to have custom-built AI solutions.

### Prospects

A USAID report highlights four key areas where MAI might play an active role in Africa, namely, population health, individual care, health systems, and pharmaceuticals and medical technology (38). These encompass roles in improving the efficiency of programs ranging from immunization, logistics, and supply chain, referral services, diagnosis, drug safety and pharmacovigilance, and performance of limited medical procedures. With the worsening shortage of health workers, it is hoped that AI will help complement inadequate human resources through telehealth. Chatbots may minimize hospital visits and assist with triaging before medical consultation. Specially designed AI mobile applications requiring little skills can help in diagnosing birth asphyxia and malaria in the rural areas of Africa where there is a shortage of skilled health workers and medical equipment.

When electronic disease surveillance becomes more prevalent; MAI may be useful in the attenuation of epidemics, particularly in Africa where disease outbreaks are recurrent. The use of AI in administrative and clinical services can help to save time on routine tasks such as automation of adjunct activities like writing chart notes, prescribing medications, and ordering tests (39). Provision of personalized and efficient treatment through AI-based analysis of thousands of medical papers or publications will provide improved and up-to-date patient care and reduce operational costs.

### Recommendations

We recommend the acceleration of the ongoing improvement in Africa's infrastructure especially electricity and Internet penetration. Reliable power supply and affordable Internet services will catalyze data generation and analysis needed for advanced automation of processes involved in patient care. Widespread use of electronic medical records and large medical databases will foster machine learning that is programmable for Africa.

By increasing the number of data scientists in Africa, a sizable workforce of experts will be made locally available to repurpose already existing AI for medical use. Citadels of learning should be adequately funded with more focus on bioinformatics, data science, computational biology, genomics, and applied mathematics. The field may become more attractive to prospective students if graduates of data science and related courses have better work opportunities in the marketplace. It is therefore imperative for private organizations to partner with educational institutions by providing internships before graduation.

With smartphone penetration projected to increase by over 100% in Africa over the next 30 years, AI developers need to put more focus on solutions that can be easily deployed on smartphones rather than on personal computers (40). Smartphones are cheaper, easier to power, and more portable for the myriads of health workers across the continent. Governments in African countries need to eschew disunity. By furthering cooperation between governments and states, trade, and commerce will grow, insecurity will be surmounted, and more resources will be available to create more robust, digitalized healthcare for the continent.

## Conclusion

There is tremendous promise in the possibilities that AI offers in transforming and improving healthcare in low-resource areas like Africa. The existing use cases show that it is a viable tool for tackling health challenges, reducing costs, and improving health access and quality. Rather than mere enthusiasm to try out new methods, an evidence-based approach should be employed in decision-making and implementation of AI in healthcare. A major lesson from the experience of AI professionals working in resource-poor settings is that AI implementation should focus on building intelligence into existing systems and institutions rather than attempting to start from scratch or hoping to replace existing systems. African countries must also enact laws and policies that will guide the application of this technology to healthcare and protect the users.

## Author Contributions

AOw wrote the initial draft and worked on the literature search. JO contributed majorly to the writing of the technical challenges' aspect of the article. AOs contributed to writing all aspects of the article and edited the initial drafts. AB reviewed, edited, and made contributions on different aspects of the article. All authors contributed to the article and approved the submitted version.

## Conflict of Interest

JO was employed by the company ZMP Inc., Japan. The remaining authors declare that the research was conducted in the absence of any commercial or financial relationships that could be construed as a potential conflict of interest.
